# Quantitative hormone therapy follow-up in an ER+/ERαKD mouse tumor model using FDG and [^11^C]-methionine PET imaging

**DOI:** 10.1186/2191-219X-2-61

**Published:** 2012-11-09

**Authors:** Michel Paquette, Sébastien Tremblay, Francois Bénard, Roger Lecomte

**Affiliations:** 1 Sherbrooke Molecular Imaging Center, Department of Nuclear Medicine & Radiobiology, Faculty of Medicine and Health Sciences, Université de Sherbrooke, 3001, 12th Avenue N., Sherbrooke, Québec, J1H 5N4, Canada; 2 BC Cancer Agency Research Center, Department of Radiology, University of British Columbia, Vancouver, British Columbia, V5Z 1L8, Canada

**Keywords:** Breast cancer, Tumor mouse model, Estrogen receptor α, Hormone therapy, Small animal PET, FDG, [^11^C]-methionine, Quantitative PET

## Abstract

**Background:**

The estrogen receptor α (ERα) is known to play an important role in the modulation of tumor response to hormone therapy. In this work, the effect of different hormone therapies on tumors having different ERα expression levels was followed up *in vivo* in a mouse model by PET imaging using 2-deoxy-2-[^18^F]fluoro-d-glucose (FDG) and [^11^C]-methionine ([^11^C]-MET). A new model of MC7-L1 ERα-knockdown (ERαKD) tumor cell lines was designed as a negative estrogen receptor control to follow up the effects of changes in ERα expression on the early metabolic tumor response to different hormone therapies.

**Methods:**

MC7-L1 (ER+) and MC7-L1 ERα-knockdown cell lines were implanted subcutaneously in Balb/c mice and allowed to grow up to 4 mm in diameter. Animals were separated into 4 groups (*n* = 4 or 5) and treated with a pure antiestrogen (fulvestrant), an aromatase inhibitor (letrozole), a selective estrogen receptor modulator (tamoxifen), or not treated (control). Tumor metabolic activity was assessed by PET imaging with FDG and [^11^C]-MET at days 0 (before treatment), 7, and 14 after the treatment. Tumor uptake of each radiotracer in %ID/g was measured for each tumor at each time point and compared to tumor growth. Quantitative PCR (qPCR) was performed to verify the expression of breast cancer-related genes (ERα, ErbB2, progesterone receptor (PR), and BRCA1) in each tumor cell lines.

**Results:**

While both ER+ and ERαKD tumors had similar uptake of both radiotracers without treatment, higher uptake values were generally seen in ERαKD tumors after 7 and 14 days of treatment, indicating that ERαKD tumors behave in a similar fashion as hormone-unresponsive tumors. Furthermore, the ERα-specific downregulation induced a slight PR expression decrease and overexpression of BRCA1 and ErbB2.

**Conclusion:**

The results indicate that the proposed ER+/ERαKD tumor-bearing mouse model is suitable to test pure antiestrogen and aromatase inhibitor therapies *in vivo* in a preclinical setting and could help to elucidate the impact of ERα levels on tumor response to hormone therapy.

## Background

Hormone therapy has been successfully used to treat estrogen receptor positive (ER+) breast cancer for a few decades. About 70% of all breast cancers are ER+ and, thus, potentially sensitive to hormone therapies [1]. However, loss of positivity, reduction of the receptor expression, and/or loss of estrogen growth dependence (to name a few) can sometimes induce resistance to hormone therapy [2,3]. Other mechanisms of resistance were proposed, such as overexpression and crosstalk of growth factors and growth factor receptors with ER pathways [4-6]. Also, a significant proportion of ER+ tumors fails to respond to some or all hormone treatments [1,7]. Hence, the contribution of estrogen receptor expression levels to the mechanisms of resistance is yet to be elucidated. Moreover, with the development of new hormone therapy agents, and data yet missing on existing therapies, a suitable animal model to test the potential of these treatments is lacking but would be essential for a better understanding of the ER role in hormone therapy.

To evaluate the impact of relatively different estrogen receptor α (ERα) expression on hormone treatments, MC7-L1 cells received a shRNA sequence targeting specifically the ERα mRNA using a lentiviral vector, thus creating an MC7-L1 ERα-knockdown (ERαKD) cell line with roughly 50% to 60% drop in ER expression. These cell lines have been previously described and characterized [8,9]. MC7-L1 tumors are already known to respond to different hormone treatments [10,11]. The comparative follow-up of ER+ and ERαKD tumors could therefore become a powerful preclinical tool to test new drugs targeting the estrogen receptors.

Since there are three main classes of estrogen hormone therapy (pure antiestrogen, aromatase inhibitor, and selective estrogen receptor modulator (SERM)), it was deemed important to test the proposed ER+/ERαKD tumor-bearing mouse model with at least one well-known drug of each class. Hence, fulvestrant, letrozole, and tamoxifen have been used in this study. With each class of drug having a different mechanism of action (that is, ER antagonist, estrogen synthesis inhibitor, and partial ER agonist, respectively), the study will provide a good indication of whether or not this ER+/ERαKD model has a universal potential in testing hormone treatments.

Small animal positron emission tomography (PET) imaging allows the non-invasive follow-up of a number of conditions in a preclinical setting. It has been used to study the expression of estrogen receptors [9,10] as well as to assess the effect of chemotherapy and hormone therapy in mammary carcinoma tumor models [11]. In the present study, 2-deoxy-2-^18^F]fluoro-d-glucose (FDG) and ^11^C]-methionine (^11^C]-MET) were chosen for the complementary data they supply on the metabolic state for the fate of the tumor. While FDG is a well-known glucose analog tracer, ^11^C]-MET is mainly incorporated into the newly synthesized proteins of tumor cells. A proliferation tracer such as 3^′^-deoxy-3^′^-^18^F]fluorothymidine (^18^F]-FLT) would have been another option to assess tumor growth rate, but thymidine analogs are known to have a poor specific uptake in rodent models without *in vivo* enzymatic degradation of endogenous thymidine [12,13], or the use of human tumors in a nude mouse model [14,15]. However, since protein synthesis is at a peak at the S-phase of the cell cycle, ^11^C]-MET uptake has been reported to somewhat correlate with the Ki67 cell proliferation index [16], proliferating cell nuclear antigen index [17], and S-phase fraction of cell population [18]. Hence, the ^11^C]-MET uptake can be used as an indirect, yet useful, indicator of the proliferative state of a tumor at a given time in rodent models.

In this study, the novel ER+/ERαKD tumor-bearing mouse model was investigated by PET imaging as a preclinical tool to follow up hormone therapies. Three different classes of estrogen hormone therapy were monitored in comparison to controls for their effect on the short-term metabolic response of tumors using both FDG and [^11^C]-MET PET scans. In parallel, a preliminary comparison of the gene expression patterns in ER+ and ERαKD tumors was performed by quantitative PCR (qPCR) to better characterize the different tumor cell lines.

## Methods

### Cell line modification

The human cell line 293T received via lipofectamine transfection 7.5 μg of the plp1, plp2, and plp/VSV-G plasmids (Invitrogen, Carlsbad, CA, USA) and 7.5 μg pLKO.1-puro plasmid containing a shRNA sequence targeting the murine ERα (Sigma-Aldrich Corporation, St. Louis, MO, USA). After 48-h incubation, the lentivirus-rich supernatant (cell media) was taken and filtered with a 0.45-μm filter then kept at −80°C for further use.

MC7-L1 cell line (murine mammary ductal carcinoma, ER+, described in [8]) was infected by an aliquot of the virus-enriched supernatant containing 4 μg/ml polybrene. The next day, cells were incubated for at least 1 week in DMEM containing 3 μg/ml of puromycin as the selection agent. Puromycin-resistant cells were expanded and further tested to see if the expression of the ERα gene was knocked down (ERαKD). Characterization of the ER status of the two cell lines by Western blot, qPCR, ^3^H]-estradiol saturation curves, and 16α-^18^F]fluoro-17β-estradiol PET imaging were described earlier [9]. All manipulations were performed following containment level 2 procedures.

### qPCR

Expression levels of ERα, BRCA1, ErbB2, and PR mRNA were obtained by real-time PCR. Total RNA extractions were performed on cell pellets with the RNeasy mini kit (Qiagen, Valencia, CA, USA) as recommended by the manufacturer, with DNAse treatments. RNA integrity was assessed with an Agilent 2100 Bioanalyzer (Agilent Technologies, Inc., Santa Clara, CA, USA). Reverse transcription was performed on a maximum of 2 μg total RNA with transcriptor reverse transcriptase, random hexamers, dNTPs (Roche Diagnostics, Basel, Switzerland), and 10 units of RNAseOUT (Invitrogen) following the manufacturer’s protocol in a total volume of 20 μl. All forward and reverse primers were individually resuspended to 20- to 100-μM stock solution in Tris-EDTA buffer (IDT) and diluted as a primer pair to 1 μM in RNase DNase-free water (IDT). qPCR reactions were performed in 10 μl in 96 well plates on a Realplex 2 thermocycler (Eppendorf, Westbury, NY, USA) with 5 μl of 2X FastStart Universal SYBR Green Master mix (Roche Diagnostics), 10 ng (3 μl) cDNA, and 200 nM final (2 μl) primer pair solutions. The following cycling conditions were used: 10 min at 95°C; 50 cycles: 15 s at 95°C, 30 s at 60°C, and 30 s at 72°C. Relative expression levels were calculated using the qBASE framework [19] and the housekeeping genes UBC, HPRT1, and GAPDH for mouse cDNA. Primer design and validation were evaluated as described elsewhere [20]. In every qPCR run, a template free control was performed for each primer pair, and these were consistently negative.

Relative quantification was achieved by attributing the arbitrary value of 1.0 to one of the three ER+ samples for each gene; the 2 other ER+ samples and the 3 ERαKD samples were then compared to this value. Average ± standard deviation of each triplicate was then used to express the relative expression level of ER+ and ERαKD cell lines for each monitored gene.

### Animals

The animal experiments were conducted according to the recommendations of the Canadian Council on Animal Care and the in-house Ethics Committee for Animal Experiments. MC7-L1 tumors (ER+ and ERαKD) were inoculated subcutaneously (1 × 10^7^ cells) in the axillary area of Balb-c mice (Charles River, Montreal, Canada). Implantation of the tumors was performed under anesthesia (13 mg/ml ketamine, 86 mg/ml xylazine; 1 ml/kg, i.p.). The tumors were grown up to 3 to 4 mm in diameter (21 to 25 days post-implantation) before initiating the PET scan schedule together with the different treatment regimens.

### Treatment regimen

One group of tumor-bearing mice received a unique subcutaneous 0.5-mg injection of fulvestrant (Sigma-Aldrich, I4409) immediately after the end of the day 0 PET scan. A *per diem* oral administration of 5 mg/kg letrozole (Femara pills (Novartis Pharmaceuticals Canada Inc., Dorval, Canada) containing 2.5 mg letrozole, dissolved in appropriate volume) was given to a second group of mice from day 1 to day 14. A third group received after the first scan a *per diem* oral dose of 8 mg/kg tamoxifen (Sigma-Aldrich, T5648). Finally, a control group without treatment served to monitor the normal *in vivo* growth and radiotracer uptake of both types of tumors. Tumor growth was monitored by caliper measurements at days 0, 7, and 14 and calculated in mm^3^ using the formula 0.524 × (width)^2^ × length for each group [21].

### Radiochemistry

Fluorine-18 was prepared by the ^18^O(p,n)^18^F reaction on ^18^O-enriched water as target material using a TR-19 cyclotron (ACSI, Vancouver, Canada). ^11^C]-carbon dioxide was prepared by the ^14^N(p,α)^11^C reaction on a gas mixture of 99.5% nitrogen and 0.5% oxygen as target, also using the TR-19 cyclotron. The methods used for the synthesis of FDG [22] and ^11^C]-MET [23] have been described elsewhere.

### PET imaging

The mice, under isoflurane anesthesia (1% at 2 l/min oxygen flow) for at least 30 min, were placed in prone position on the bed of a LabPET™ small animal PET scanner (Gamma Medica, Northridge, CA, USA), having a 3.75-cm axial field of view and achieving 1.35-mm resolution [24]. One group of mice for each treatment regimen (and the untreated control group) was imaged 30 min after the i.v. injection (via the caudal vein) of 20 to 24 MBq ^18^F]-FDG. Image acquisition was performed for 15 min with the tumors centered in the scanner field of view. Another group received an injection of 20 to 24 MBq ^11^C]-MET in the caudal vein, and 10 min later, the mice were imaged during 20 min. All image acquisitions were performed with dual axial sampling positions to improve image uniformity and resolution in the axial direction. Due to logistic constraints, two different groups were used for either FDG or ^11^C]-MET imaging.

The images were reconstructed using 20 iterations of a 2D MLEM algorithm implementing a physical description of the detector responses in the system matrix [25]. Quantification of the tumor uptake was performed using the in-house LabTEP image analysis software. The FDG and ^11^C]-MET signals were estimated by searching the highest 2 × 2 cluster of voxels within the tumor ROI. The background from circulating radiotracer and non-specific tissue uptake was estimated from a reference ROI placed on the muscular tissues. This average background count rate was subtracted from the tumor peak count rate to obtain the net tumor uptake. After each scan sequence, a cylindrical phantom that approximates the size of a mouse (24.8 ml, 26-mm diameter × 47-mm axial length) containing a known quantity of ^18^F (≈20 MBq FDG) was used to obtain a calibration factor to convert the counts per second into absolute activity measurements in kilobecquerel, from which the injected dose per gram of tissue (%ID/g) values were derived. A density of 1 g/cc was used to convert the fractional uptake per volume into %ID/g. The uptake values were not corrected for partial volume averaging effects as the tumor size was above the threshold (>3-mm diameter or >14-mm^3^ sphere) for which recovery correction factors become necessary using the LabPET™ scanner.

### Statistical analysis

Standard deviation of the mean was used to determine the spread of data around the mean. Although the data sets are relatively small (four or five per group), it is assumed that they follow a normal distribution. Therefore, a paired two-tailed Student’s *t* test was used to evaluate uptake differences between ER+ and their corresponding (in the same animal) ERαKD tumors using a probability threshold of *p* = 0.05 for significance. For comparison between the different treatment groups and the control group, an unpaired two-tailed Student’s *t* test was used, with a probability threshold of *p* = 0.05.

## Results

### Tumor growth during treatment

**Figure 1:**
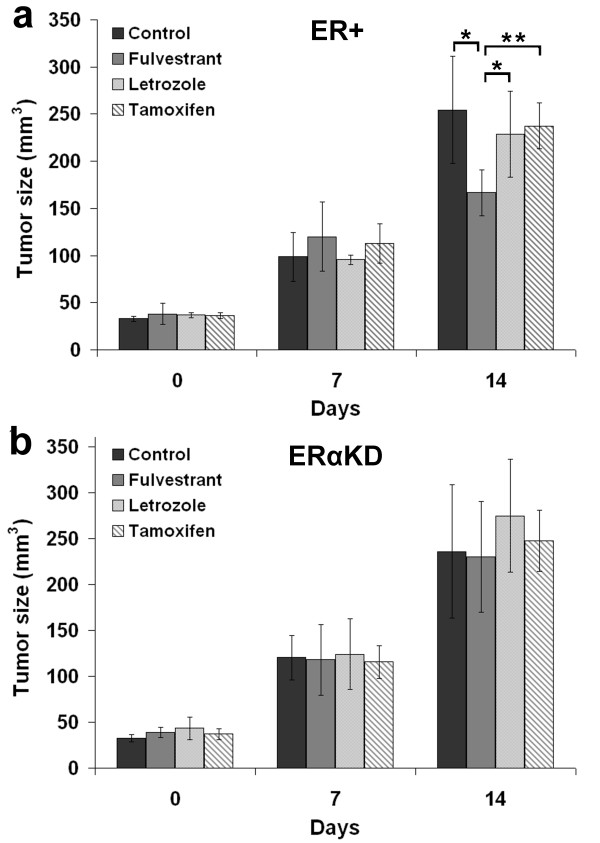
**Caliper growth follow-up measurements.** MC7-L1 ER+ (**a**) and ERαKD (**b**) tumor growth were measured under fulvestrant, letrozole, or tamoxifen therapy, or no treatment (control). Measurements were done at 0, 7, and 14 days following the same time points as the imaging protocol (*n* = 5 for each group). Asterisk denotes *p* < 0.05; double asterisk, *p* < 0.01.

Caliper measurements were taken at each time point for each treatment group to assess the effects of the hormone therapies on the growth rate of the two different tumors during the short (14 days) time scale of the study (Figure 1). While both tumor types grew at a very similar rate in the absence of treatment (control group), or under letrozole or tamoxifen treatment, ER+ tumors grew significantly slower than ERαKD tumors after 14 days of fulvestrant treatment (*p* < 0.05). Letrozole treatment caused a non-significant trend (*p* = 0.07) towards growth inhibition of ER+ tumors compared to ERαKD tumors after 14 days. Moreover, MC7-L1 tumors treated with fulvestrant for 14 days had their growth significantly inhibited compared to the control (*p* < 0.05), letrozole (*p* < 0.05), and tamoxifen (*p* < 0.01) groups at 14 days. The other treatments failed to differentiate the growth of either tumors compared to control (*p* > 0.21). Attempts to follow tumor growth for a longer time resulted in the animals reaching endpoints (partial paralysis, growth-related necrosis of the tumor, etc.) well before day 21. Under therapy, ERαKD tumors were the cause for reaching endpoints most of the time.

### Comparative qPCR

**Figure 2:**
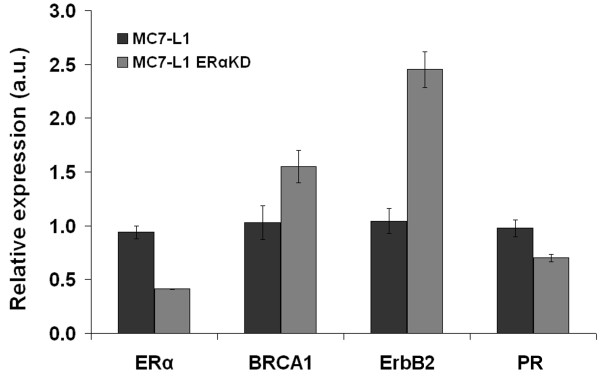
**Comparative qPCR.** Murine ERα, BRCA1, ErbB2, and PR mRNA expression were compared in MC7-L1 ER+ and ERαKD cell lines. RNA extraction was performed in triplicate for each cell line, and each qPCR reaction (each sample, each gene) was repeated thrice. Data were expressed relative to one of the three MC7-L1 samples for each gene.

Comparative qPCR of ER+ and ERαKD cell lines was performed on a sample of relevant genes (ErbB2, BRCA1, PR, and of course ERα) to verify if the specific knockdown of ERα affected the expression of other breast cancer-related genes (Figure 2). The shRNA-dependent 60% drop of the ERα in the ERαKD cell line (as compared to ER+, *p* < 0.0001) is accompanied by a slight but significant decrease of estrogen-dependent PR expression (*p* < 0.005) and also by a 1.5-fold increase of BRCA1 mRNA levels (*p* < 0.05) and by a 2.5-fold rise of ErbB2 mRNA levels (*p* < 0.0005).

### FDG PET images and quantification

**Figure 3:**
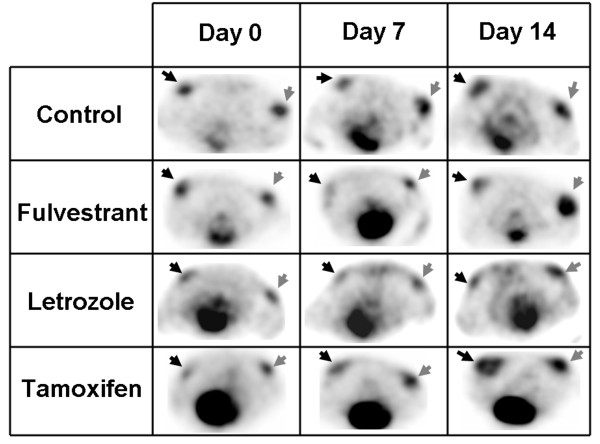
**Representative transaxial slices of FDG PET images.** Images were taken at days 0, 7, and 14 after the start of a treatment regimen (control, 0.5 mg fulvestrant, 5 mg/kg/day letrozole, and 8 mg/kg/day tamoxifen). Black arrows indicate ER+ tumors; gray arrows, ERαKD tumors.

**Figure 4:**
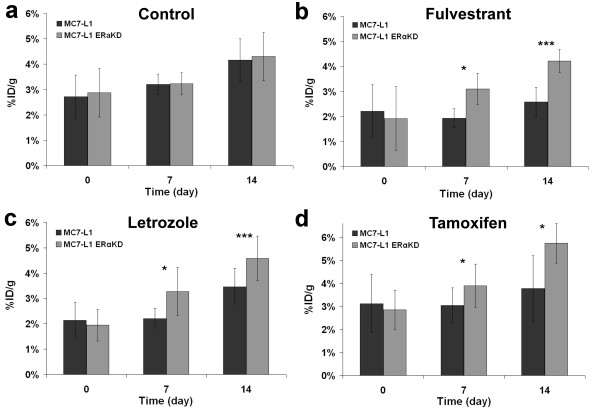
**Quantitative measurements of FDG tumor uptake (expressed in %ID/g).** Uptake in MC7-L1 ER+ and ERαKD tumor-bearing mice was measured at days 0, 7, and 14 after the start of the treatment (control, *n* = 5 (**a**); fulvestrant, *n* = 4 (**b**); letrozole, *n* = 5 (**c**); tamoxifen, *n* = 5 (**d**)). Asterisk denotes *p* < 0.05; triple asterisk, *p* < 0.005.

Selected results of the 14-day follow-up study performed using FDG on ER+ and ERαKD tumors in the different groups (control, fulvestrant, letrozole, and tamoxifen) are shown in Figure 3. For the control group (*n* = 5), the uptake increased gradually with time, and no significant differences in the uptake value (*p* > 0.33) were seen between ER+ and ERαKD tumors (Figure 4a).

With the fulvestrant treatment (*n* = 4, Figure 4b), significant uptake differences were observed by FDG PET imaging between ER+ and ERαKD tumors at day 7 (*p* < 0.05) and day 14 (*p* < 0.005), whereas the same trend (*p* < 0.05 at day 7, *p* < 0.005 at day 14) was observed using letrozole (*n* = 5, Figure 4c). For the tamoxifen group (*n* = 5, Figure 4d), ERαKD tumors had a statistically significant higher uptake than ER+ tumors at days 7 and 14 (both at *p* < 0.05).

### [^11^C]-MET PET images and quantifications

**Figure 5:**
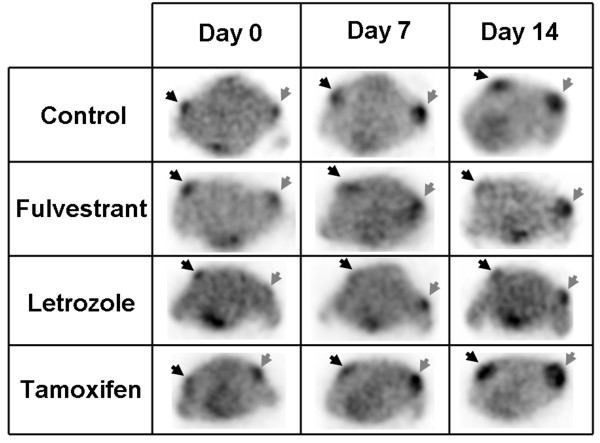
**Representative transaxial slices of [**^**11**^**C]-MET PET images.** Images were taken at days 0, 7, and 14 after the start of a treatment regimen (control, 0.5 mg fulvestrant, 5 mg/kg/day letrozole, and 8 mg/kg/day tamoxifen). Black arrows indicate ER+ tumors; gray arrows, ERαKD tumors.

**Figure 6:**
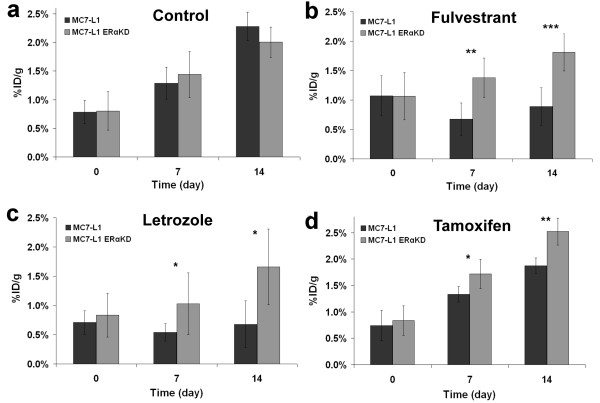
**Quantitative measurements of [**^**11**^**C]-MET tumor uptake (expressed in %ID/g).** Uptake in MC7-L1 ER+ and ERαKD tumor-bearing mice was measured at days 0, 7, and 14 after the start of the treatment (control, *n* = 5 (**a**); fulvestrant, *n* = 5 (**b**); letrozole, *n* = 4 (**c**); tamoxifen, *n* = 5 (**d**). Asterisk denotes *p* < 0.05; double asterisk, *p* < 0.01; and triple asterisk, *p* < 0.005.

Parallel to the FDG study, the 14-day follow-up of the different treatment groups was performed using [^11^C]-MET PET imaging (Figure 5). As with FDG, the [^11^C]-MET control group (*n* = 5) showed a progressive increase of uptake between day 0 and day 14, with both ER+ and ERαKD tumors having no significant uptake differences (*p* > 0.16) throughout the follow-up period (Figure 6a).

[^11^C]-MET uptake was clearly higher in ERαKD tumors, as compared to ER+ tumors, at day 7 (*p* < 0.01) and day 14 (*p* < 0.005) during the course of the fulvestrant treatment (*n* = 5, Figure 6b). Similarly, the follow-up of letrozole therapy (*n* = 4, Figure 6c) using [^11^C]-MET PET also showed a significant uptake difference favoring ERαKD tumors at day 7 and day 14 (both at *p* < 0.05). Following the same trend, ERαKD tumors had a significantly higher uptake than ER+ tumors at day 7 (*p* < 0.05) and day 14 (*p* < 0.01) during the tamoxifen treatment (*n* = 5, Figure 6d).

### Uptake of treated versus untreated groups

**Figure 7:**
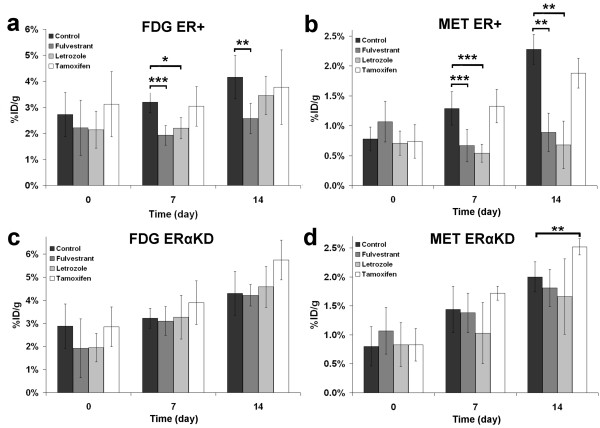
**Comparison of %ID/g uptake in the control group and the three treatment groups.** Uptake was compared for ER+ tumors imaged with FDG (**a**) and [^11^C]-MET (**b**) and for ERαKD tumors imaged by PET with FDG (**c**) and [^11^C]-MET (**d**). Asterisk denotes *p* < 0.05; double asterisk, *p* < 0.01; and triple asterisk, *p* < 0.005.

In order to assess the therapeutic effect of the different medications tested on both types of tumors, the uptake of each treated group was compared to the values from the untreated group obtained with the same tracer. Hence, for the FDG measurements of the ER+ tumor response (Figure 7a), the uptake was significantly lower than the control group for fulvestrant at day 7 (*p* < 0.005) and day 14 (*p* < 0.01), and for letrozole at day 7 (*p* < 0.05). A near-significant trend towards lower than the control uptake could be seen at day 14 of letrozole therapy for ER+ tumors (*p* = 0.067). As for tamoxifen treatment, no difference was observed for ER+ tumor compared to the control group (day 7, *p* = 0.33; day 14, *p* = 0.48). On the other hand, ERαKD tumors under fulvestrant and letrozole therapies, followed-up by FDG (Figure 7c), were undistinguishable from the control group (*p* varying between 0.30 and 0.85), although there was a non-significant trend towards a higher uptake than the control for tamoxifen therapy (*p* = 0.08 for day 7, *p* = 0.06 for day 14).

Using [^11^C]-MET measurements of tumor response, a similar pattern to FDG was observed. Indeed, ER+ tumors (Figure 7b) treated with fulvestrant had a lower uptake than the control group (*p* < 0.005 at day 7, *p* < 0.01 at day 14). Similar results were observed using letrozole (*p* < 0.005 at day 7, *p* < 0.01 at day 14). Again, tamoxifen did not affect the [^11^C]-MET uptake of ER+ tumors compared to the untreated group (day 7, *p* = 0.42; day 14, *p* = 0.31). For ERαKD tumors (Figure 7d), there was no significant [^11^C]-MET uptake differences between the treated and untreated groups (*p* varying between 0.12 and 0.39), with the exception of tamoxifen at day 14, where a higher than control uptake is observed (*p* < 0.01).

## Discussion

In this study, the short-term (14 days) follow-up of three different estrogen hormone therapies on a novel ER+/ERαKD mouse tumor model was performed by means of FDG and [^11^C]-MET PET imaging. Each of these treatments represents one of the three main classes of hormone therapy, each class having a different mechanism of action (partial agonist, pure antagonist, or aromatase inhibitor). At the same time, the use of FDG and [^11^C]-MET allowed different information related to the glycolytic activity and protein synthesis rate to be obtained to better characterize the tumor fate.

The sensitivity of MC7-L1 tumors to tamoxifen and letrozole hormone therapies (among other therapies) was already evaluated by caliper measurements in a previous study [10]. A growth inhibition was observed after 6 to 7 weeks of treatment, as compared to the untreated group, with the therapies beginning at the time of implantation of the tumors. It was then concluded that MC7-L1 tumors were responsive to hormone therapy treatments. Another study followed up letrozole treatment and chemotherapy on MC7-L1 and MC4-L2 tumor-bearing mouse models using FDG PET [11]. However, tumors were grown up to at least 6-mm diameter (≈100 μl) before the start of the treatments, and evident signs of necrosis were seen on PET images during follow-up and, in some cases, even at day 0. A more recent investigation using spontaneously occurring mammary STAT1 −/− tumors in a mouse model employed FDG and steroid receptor PET imaging to follow up hormone therapies [26]. Using this model, a significant drop in FDG tumoral uptake was observed 2 weeks after administration of 5 mg/week of fulvestrant (a tenfold higher dosage), with treatment beginning 23 days after tumor implantation. In contrast to our study, this work was mainly focused on the assessment of steroid receptor modulation by PET under hormone therapy.

In the present work, treatments began at 21 to 25 days after tumor implantation, hence more representative of a therapeutic protocol than a prophylactic or adjuvant setting. In these conditions, follow-up could hardly be pursued for a longer time period than 14 days because endpoints were reached (most of the time, due to ERαKD tumors). Besides, tumors would begin to show signs of necrosis, which could have influenced tumor uptake for other reasons than treatment efficacy. However, a short-term reduction of FDG and [^11^C]-MET uptake (after 7 and 14 days of treatment) was clearly observed in the MC7-L1 ER+ tumor when using fulvestrant or letrozole treatment compared to the control group (with the exception of letrozole followed by FDG at day 14, where a non-significant reduction was observed). Moreover, both tracers succeeded in differentiating the ER+ tumor from the ERαKD tumor at days 7 and 14, regardless of the treatment used.

On the other hand, the ERα-knockdown variant of the MC7-L1 cell line did not have such uptake inhibition when under therapy, which could be the direct result of ERα downregulation. Nevertheless, other studies suggest that one of the main factors responsible for hormone therapy resistance is the overexpression of EGFR and ErbB2 (Her2), which not only can crosstalk with ERα signaling [27] but also can act as a compensation mechanism [28]. For instance, letrozole-resistant MCF-7 tumors were reported to have a fourfold increase in ErbB2 expression compared to control tumors [29]. Moreover, combination therapy using both letrozole and transtuzumab in an aromatase-transfected MCF-7 xenograft model reversed letrozole resistance and sensitized the tumors to estrogen, further supporting the role of Her2 in hormone resistance [28]. Interestingly, the ERα-specific knockdown in our MC7-L1 cell line provoked a 2.5-fold increase in ErbB2, which could also be another reason why the ERαKD tumors resisted hormone treatments (as assessed by FDG and ^11^C]-MET uptakes).

Tamoxifen therapy gave ambiguous results: on one hand, FDG and ^11^C]-MET uptakes were significantly lower in wild-type MC7-L1 tumors than in their ERαKD counterpart after 7 and 14 days of treatment, hence supporting that these tumors reacted differently under hormone therapy. On the other hand, there were no significant differences between the uptake of the tamoxifen group and the control group, with the exception of ^11^C]-MET, day 14, where ERαKD tumors had actually higher uptake than the control group. Tamoxifen therapy is known to induce a short-term metabolic flare, a phenomenon already reported in FDG PET follow-up studies [30], which could well be observed in the present study. Hence, although the tamoxifen dose (8 mg/kg/day) used in this study was found optimal for growth inhibition of the MC7-L1 tumor in a previous study [10] and other doses tested in the present study were either ineffective (4 mg/kg/day) or growth- and uptake-stimulating (16 mg/kg/day, data not shown), it can be concluded that this model and methodology are limited in their capacity to evaluate SERM therapies on a short time scale.

FDG and ^11^C]-MET PET imaging was successful in distinguishing ER+ from ERαKD tumors treated with the different hormone therapies and in assessing early treatment efficacy of ER+ tumors for fulvestrant and letrozole in most cases. It is noteworthy that a glucose analog tracer and a protein synthesis/amino acid transport tracer uptake both follow the same trend throughout the different therapies. On the other hand, this is not surprising, considering that a clinical study using FDG and ^11^C]-MET PET with various types of tumors has shown a good correlation (*R* = 0.79) between the uptake of these two tracers [31]. Not unexpectedly, growth follow-up of these tumors was much less successful in monitoring an effect of these therapies on such a short time scale. With the exception of fulvestrant on day 14, where a significant size difference was observed between ER+ and ERαKD tumors and between treated and untreated ER+ tumors, no other effect could be observed using caliper measurements. On a longer time scale, it is already known that letrozole and tamoxifen induce a growth inhibition for treated compared to untreated MC7-L1 tumors [10]. Our results support the fact that an earlier evaluation of therapy success than growth follow-up can be obtained with FDG and ^11^C]-MET PET, at least for the fulvestrant and letrozole treatments.

Interestingly, despite the fact that the ERα-specific downregulation induced varying expression patterns in the studied genes (and probably in other unmonitored genes), MC7-L1 ERαKD displayed a phenotype that was very similar to the parental cell line. Indeed, the morphology of the cells, the uptake of metabolic PET tracers, and the *in vivo* and *in vitro* growth rates were all comparable between the two cell lines, with the notable phenotypic exception of how they withstand hormone therapy. To explain this phenotype, it seems likely that the residual ERα activity, together with the contribution of ERβ activity, was sufficient to maintain the estrogen signaling pathways at a suitable level to allow normal growth. Alternatively, ErbB2 overexpression could also somewhat compensate for the partial loss of ERα.

Finally, the ER+/ERαKD mouse tumor model, combined with FDG and ^11^C]-MET PET imaging, would represent a valuable test bench for new ER-specific therapies [32,33] or for optimizing the dose regimen and administration protocols of existing antiestrogen or aromatase inhibitor therapies. Even though no clear outcome of tamoxifen treatments could be demonstrated, the proposed tumor model could still be useful to investigate the SERM action mechanisms. Moreover, it could also be used to test whether new treatments and protocols are effective against hormone therapy-resistant tumors [27]. In parallel, wider and more detailed gene expression comparisons would help to better characterize ER+ and ERαKD cell lines.

## Conclusion

Using a novel ER+/ERαKD murine breast tumor model, different estrogen hormone therapies were evaluated longitudinally (on a short-term 14-day schedule) using FDG and [^11^C]-MET PET imaging. With this new model, it was possible to observe that letrozole and fulvestrant treatments reduced glucose uptake/consumption and protein synthesis in ER+ tumors, but not so in ERαKD tumors, on this short time scale. Although tamoxifen treatment showed differences in response between both tumor types, comparison with the control group was inconclusive. Altogether, the proposed ER+/ERαKD tumor-bearing mouse model provides a promising preclinical platform to investigate novel ER-specific therapies using PET imaging.

## Competing interests

The authors declare that they have no competing interests.

## Authors’ contributions

MP laid out the study design, carried out the experimental procedures, performed the statistical analysis, and drafted the report. ST carried out the synthesis of [^11^C]-methionine and participated in the coordination of the project. FB and RL participated in the design and coordination of the study and helped draft and correct the manuscript. All authors read and approved the final manuscript.
